# Native T1 values in discrimination of subclinical profibrotic phenotype in relatives of patients with hypertrophic cardiomyopathy

**DOI:** 10.1186/1532-429X-16-S1-P241

**Published:** 2014-01-16

**Authors:** Rocio Hinojar, Benjamin P Goodman, Adriana Villa, Darius Dabir, Eduardo Arroyo Ucar, Thomas Jackson, Tobias Schaeffter, Eike Nagel, Valentina Puntmann

**Affiliations:** 1 Cardiovascular Imaging Department, King's College London, London, UK

## Background

Hypertrophic cardiomyopathy (HCM) is associated with significant associated morbidity and mortality. Increased maximal left ventricular wall thickness (LVWT) has been postulated as major risk factor of sudden death; however, relatives with normal LVWT are also at risk. Genetically driven interstitial collagenesis has been proposed as a possible mechanism of diffuse myocardial fibrosis and increased extracellular volume fractions (ECV) has been demonstrated in genotype positive subjects. We investigated whether native T1 can also separate genotype positive subjects in the absence of increase in LVWT and how does it relate to ECV measurements.

## Methods

Seventeen genotype positive first-degree relatives of HCM patients, and seventeen healthy volunteers underwent assessment of T1 mapping, function and scar by CMR at 3-Tesla scanner. T1 values were measured conservatively within the septal myocardium in a midventricular short-axis slice prior to and 15-20 minutes after administration of 0.2 mmol/kg of gadobutrol.

## Results

Relatives of HCM patients were well matched for age, gender and traditional cardiovascular risk factors. The groups did not differ in the conventional LV parameters, including volumes, left and right ejection fraction or LV mass. None of the studied subjects showed late gadolinium enhancement. Compared to controls, T1native values were increased in HCM relatives (control vs. relatives, T1native (msec) 1045 ± 17 vs. 1104 ± 16, p < 0.0001), whereas T1postcontrast and lambda did not vary between groups. T1native was identified as the independent discriminator to differentiate between relatives of HCM patients and controls.

## Conclusions

We demonstrate that T1native values are increased in genotype positive relatives of HCM patients. We propose that T1 native may serve as an early marker of cardiomyopathy, adding valuable information to genetic testing in this increasing population, possibly without a need for a contrast CMR study.

## Funding

We would like to acknowledge Department of Health via the National Institute for Health Research (NIHR) comprehensive Biomedical Research Centre award to Guy's & St Thomas' NHS Foundation Trust in partnership with King's College London and King's College Hospital National Health Service Foundation Trust. Dr. Rocio Hinojar was supported by the Fundacion Alfonso Martin Escudero.

**Figure 1:**
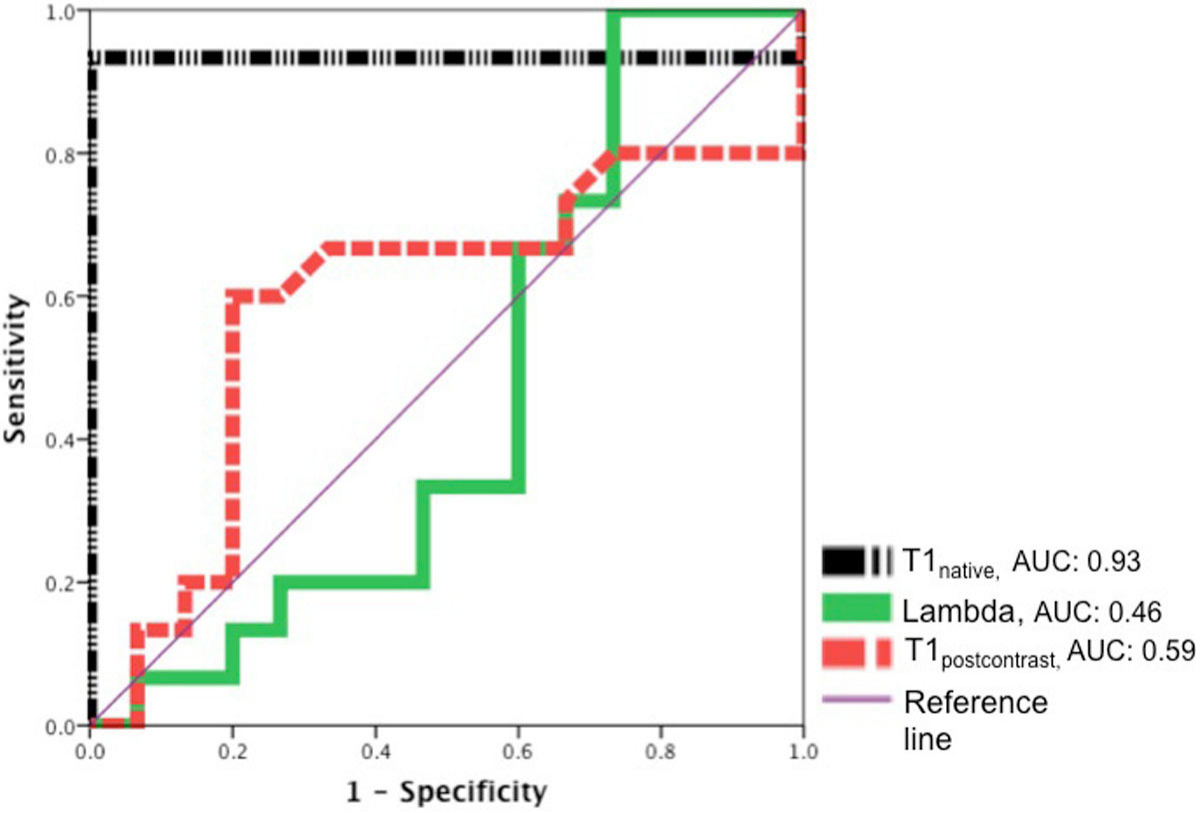
**ROC curves**. Diagnostic Performance of T1 derived indices (T1native, T1postcontrast, lambda) by T1 Mapping in the detection of genotype positive first-degree relatives of HCM patients

